# The Habit of Generalization

**Published:** 1894-04

**Authors:** 


THE HABIT OF GENERALIZATION.—When a man eats a certain article of food that disagrees with him, he immediately denounces that article of food, and his first impulse is to assume that it is injurious to *all* people. He warns everybody against it. When he is relieved of some discomfort or some pathological condition by some "home remedy," some herb, or other therapeutic measure, he becomes accordingly a warm advocate of the means that brought about the cure, and assumes that every one else must be similarly benefited by taking it.

If a person have a certain taste in dress he expects every one else who possesses *good* taste to dress similarly, and is surprised if his taste be not adopted, but a different style chosen instead. Criticisms and even game-making will quickly suggest themselves to his mind, and his lips may be tempted to utter them on observing the departure from his own standard.

This process of reasoning is called generalization.

Let us examine the definition of the word in the dictionary. For this purpose we will consult the great Standard Dictionary of Funk & Wagnalls, just published, of which a review may be found in this number at page 122:

"*Generalization.*—The process or act of generalizing; the obtaining of a general conception, rule, or law from the consideration and analysis of individual or specific cases or instances; formation of general principles or notions."

The definition here given represents what is really a natural, spontaneous, almost unconscious, and ever recurring operation of the mind, and therefore a habit.

This habit of generalizing arises out of the assumption that all people are built alike, and therefore must be affected in the same way by the same influences.

Generalizing leads a man to live like his neighbor, to imitate his personal habits, believing it must be good for himself also. Generalizing leads a man to take a quack medicine because his neighbor reports benefit from it. Generalizing leads him to believe the statements in advertisements of quack medicines, and so it is that large sales of these compounds are assured.

Generalizing creates the fabric of a pathology and a consequent scheme of treatment suitable for all people who have this pathological picture.

As just stated, it is a habit of the mind to generalize. It is common to all, with or without education, and so arises spontaneously in the mental organization of all, whenever the idea is suggested in any of the ways above enumerated. Thus it becomes a vice. It has for its companion the habit of assumption or of taking for granted that which we are inclined to believe, no matter whether we have or have not positive knowledge of the subject—in short, of *opinion*.

These two, generalization and opinion, lead us into lots of errors, divide us and our friends into parties or partisan cliques, and so erect around each individual a Chinese wall that shuts out all the world of fact and action, and keeps him a slave to his own conceptions. Here, then, we have one of the obstacles to the progress of medicine that hampers its development, and creates the criticism with which scientific literature abounds upon the "backwardness of medicine."

It must not be inferred from the foregoing that generalization is an unmitigated evil. Far from it. On the contrary, generalization has brought about the most wonderful advancement in the natural and physical sciences.

The *educated* generalization of a great mind like that of Darwin has unfolded to mankind the famous doctrine of evolution which has been such an assistance to the human mind in getting a comprehensive view and orderly arrangement of the vast array of isolated facts which everywhere abound in nature.

The *educated* generalization of Sir Humphrey Davy led him to the discovery of the alkaline and earthy metals Potassium, Sodium, Calcium, and Magnesium. When at the close of the last century the mechanism of an oxide was made plain and its combination with acids to form salts was understood, the fine mind of Davy led him to argue that the alkalies and earths, by reason of their behavior with acids, must be oxides or bases, and that in each one of these lay hidden an unknown metal. He proceeded to find this out, and accordingly took advantage of the electrolytic power of the galvanic current. With this powerful agency he tore apart the combination and the metal stood revealed, and so the problem was solved.

Frauhhofer, speculating upon the black lines of the solar spectrum—first observed but not explained by the famous Dr. Wollaston—by the aid of an educated generalization resolved them into a series of absorption effects of the light from the body of the sun passing through an incandescent atmosphere charged with the vapors of individual metals, and so arose the science of solar and stellar chemistry, and also a new method of chemical analysis in the laboratory which has since been brought to bear upon the lower potencies of the homœopathic remedies.

This kind of educated generalization may be said to have had its beginnings with Sir Isaac Newton, and to have advanced down the centuries until it reached its height in the present century, into which it was ushered by the remarkable discoveries of Davy.

These astonishing and brilliant results of generalization have had the effect of establishing unquestioning confidence in generalization and its results, and to have fixed more firmly the habit of employing it.

Now, when there comes along a system of medicine which, though in itself a generalization as far as relates to the conception of it as a law of cure, yet nevertheless in its detail almost completely ignores the idea of generalization, and compels for its successful employment a mode of thought that is the diametric opposite of the common method of thinking, such a system finds a most formidable obstacle to its further progress. And here we have one of the causes of the opposition to our system of treatment by some minds and the total incapacity to understand it of some other minds; and the whole forms a combination that holds back our school from that progress which we ought to expect from its inherent truth, our experience of it, and the analogy of the progress of the other sciences.

Thus we have found one of the principal factors in the state of scepticism which makes the obstacle to the acceptance of the homœopathic doctrine.

Much more might be said upon this theme, but this editorial has already exceeded reasonable limits, and as enough has been given to stimulate the thought of the reader, we will close.

